# (5*E*)-2-[4,5-Bis(methyl­sulfan­yl)-1,3-dithiol-2-yl­idene]-5-(4-iodo-1,3-dithiol-2-yl­idene)-1,3-dithio­lan-4-one

**DOI:** 10.1107/S1600536809044493

**Published:** 2009-10-31

**Authors:** Kazumasa Ueda, Kenji Yoza

**Affiliations:** a Division of Applied Science and Fundamental Engineering, Faculty of Engineering, Shizuoka University, Johoku 3-5-1, Hamamatsu, Shizuoka, 432-8561, Japan; b Bruker AXS Co Ltd, Moriya-cho 3-9, Kanagawa-ku, Kanagawa, Kanagawa 221-0022, Japan

## Abstract

The mol­ecular framework of the title compound, C_11_H_7_IOS_8_, is almost planar [maximum deviation = 0.040 (4) Å], except for the two methyl­sulfanyl groups, which are twisted relative to the mol­ecular skeleton, making C—S—C—C torsion angles of 144.1 (8) and −141.3 (8)°. In the crystal, mol­ecules are stacked alternately in opposite orientations, forming a one-dimensional column parallel to [110]. The primary inter­actions between mol­ecules comprising the columns are of the S⋯S type [3.554 (1) Å]. Inter­actions between columns are of the S⋯S type [3.411 (1) along *b* and 3.444 (1) Å along *c*], as well as S⋯I contacts [3.435 (2) Å].

## Related literature

For background to 2,5-di(1,3-dithiole-2-yl­idene)-1,3-dithio­lan-4-one derivatives, see: Iwamatsu *et al.* (1999 ▶); Matsumoto *et al.* (2002 ▶, 2003 ▶); Hiraoka *et al.* (2007 ▶); Ueda & Yoza (2009 ▶). For the synthesis, see: Ueda & Yoza (2009 ▶). For background to inter­molecular S⋯I contacts, see: Blake *et al.* (1997 ▶, 1998 ▶, 1999 ▶); Bricklebank *et al.* (2000 ▶); Ouvrard *et al.* (2003 ▶). For van der Waals radii, see: Bondi (1964 ▶).
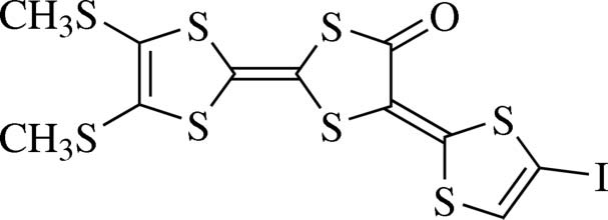


## Experimental

### 

#### Crystal data

C_11_H_7_IOS_8_
*M* *_r_* = 538.55
Triclinic, 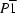

*a* = 8.309 (3) Å
*b* = 8.344 (3) Å
*c* = 14.618 (7) Å
α = 90.851 (6)°
β = 105.132 (6)°
γ = 118.510 (4)°
*V* = 848.0 (6) Å^3^
*Z* = 2
Mo *K*α radiation
μ = 2.87 mm^−1^
*T* = 93 K
0.04 × 0.04 × 0.04 mm

#### Data collection

Bruker APEXII CCD area-detector diffractometer
Absorption correction: multi-scan (*SADABS*; Sheldrick, 1996 ▶) *T*_min_ = 0.894, *T*_max_ = 0.894
9773 measured reflections
3820 independent reflections
3065 reflections with *I* > 2σ(*I*)
*R*_int_ = 0.050

#### Refinement

*R*[*F* ^2^ > 2σ(*F* ^2^)] = 0.055
*wR*(*F* ^2^) = 0.161
*S* = 1.07
3820 reflections
190 parameters
H-atom parameters constrained
Δρ_max_ = 2.34 e Å^−3^
Δρ_min_ = −1.13 e Å^−3^

### 

Data collection: *APEX2* (Bruker, 2006 ▶); cell refinement: *SAINT* (Bruker, 2006 ▶); data reduction: *SAINT*; program(s) used to solve structure: *SHELXS97* (Sheldrick, 2008 ▶); program(s) used to refine structure: *SHELXL97* (Sheldrick, 2008 ▶); molecular graphics: *Mercury* (Macrae *et al.*, 2008 ▶); software used to prepare material for publication: *XCIF* (Bruker, 2001 ▶).

## Supplementary Material

Crystal structure: contains datablocks I, global. DOI: 10.1107/S1600536809044493/tk2559sup1.cif

Structure factors: contains datablocks I. DOI: 10.1107/S1600536809044493/tk2559Isup2.hkl

Additional supplementary materials:  crystallographic information; 3D view; checkCIF report

## Figures and Tables

**Fig. 1.**
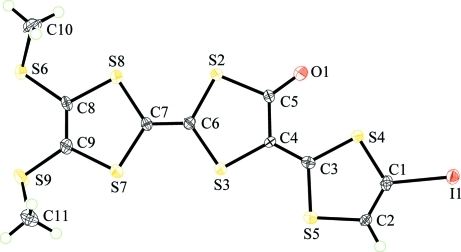
The molecular structure of (I) showing atom labeling and 50% probability of displacement ellipsoids for non H-atoms.

**Fig. 2.**
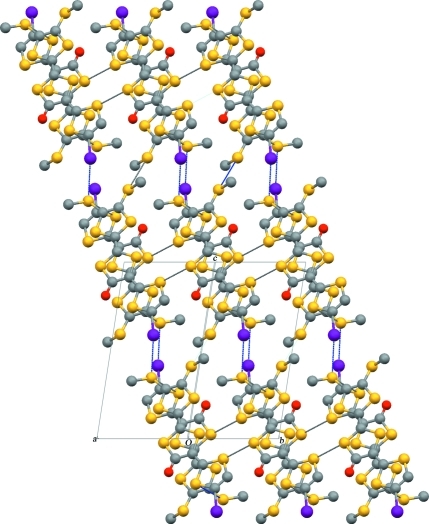
Projection of the crystal packing in (I) along the [110] direction. The S···S (gray), and S···I (blue) contacts are shown with dashed lines. H atoms are omitted for clarity.

**Fig. 3.**
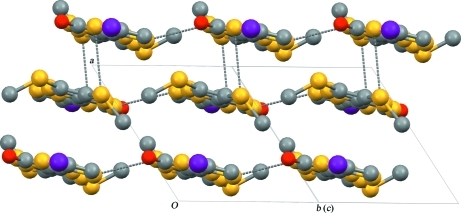
Projection of the crystal packing in (I) along the longer molecular axis. The S···S contacts are shown with gray dashed lines. H atoms are omitted for clarity.

## supplementary crystallographic information

### Comment

2,5-Di(1,3-dithiol-2-ylidene)-1,3-dithiolan-4-one derivatives are used for the preparation of charge transfer salts with magnetic metal anions (Iwamatsu *et al.*, 1999; Matsumoto *et al.*, 2002*a*, *b*, 2003; Hiraoka *et al.*, 2007). In CT salts these molecules can form unique crystal structures containing channels in addition to the usual stacked layer structures. The control of donor molecule interactions by means of chemical modification of the 2,5-di(1,3-dithiol-2-ylidene)-1,3-dithiolan-4-one skeleton may increase the dimensionality of aggregation in the solid-state. In this context, we have previously synthesized a molecule substituted with two iodide atoms, namely 2-[4,5-bis(methylsulfanyl)-1,3-dithiol-2-ylidene]-5-(4,5-diiodo-1,3-dithiol- 2-ylidene)-1,3-dithiolan-4-one, and observed fairly close I···O interactions in the crystal (Ueda & Yoza, 2009). As a continuation of these studies, herein, we present the crystal structure of a molecule substituted with one iodide atom, (I).

The molecular framework of (I), Fig. 1, except for two methylsulfanyl groups, is almost planar. The displacements of atoms S6, S9, and I1 relative to the plane of the skeleton are 0.106 (4), 0.236 (5) and 0.013 (4) Å, respectively. The torsion angles of the two methylsulfanyl groups are 144.1 (8)° for C10—S6—C8—C9 and -141.3 (8)° for C11—S9—C9—C8.

In the crystal structure, the molecules are stacked alternately in opposite orientations, forming a one-dimensional column parallel to the [110] direction (Fig. 2). The weak interactions between stacked molecules is accomplished through S···S contacts [S4···S6^i^ = 3.554 (1) Å; symmetry code (i): 2-x, 2-y, 2-z] which are shorter than the sum of van der Waals radii of two S atoms, i.e. 3.60 Å (Bondi, 1964). It is noted that although the stacked molecules are separated by interplanar distances as short as 3.54 Å, they have fairly poor overlap. Some effective side-by-side contacts are observed between molecules of adjacent columns. These interactions are accomplished through S···S contacts [S2···S5^ii^ = 3.411 (1) Å; symmetry code (ii): x, 1 + y, z] along the *b* axis. Stability along the c axis are afforded by additional S···S contacts [S9···S9^iii^ = 3.444 (1) Å; symmetry code (iii): 2 - x, 2 - y, 1 - z] as well as S···I contacts [S6···I1^iv^ = 3.435 (2) Å; symmetry code (iv): x, 1 + y, -1 + z]. This latter distance is shorter than the sum of corresponding van der Waals radii for S and I, i.e. 3.78 Å (Bondi, 1964). Such S···I interactions have been observed previously (Blake *et al.*, 1997, 1998, 1999; Bricklebank *et al.*, 2000). The intermolecular angles, 162.8 (2)° for S6···I1^iv^—C1^iv^ and 105.8 (4)° for C10—S6···I1^iv^, are close to the ideal geometry (180° for C—I···S and 109.5° for C—S···I) which have been proposed for these types of associations (Ouvrard *et al.*, 2003).

### Experimental

Compound (I) was synthesized by a modification of the method used for the preparation of 2-[4,5-bis(methylsulfanyl)-1,3-dithiol-2-ylidene]-5-(4,5-diiodo-1,3-dithiol- 2-ylidene)-1,3-dithiolan-4-one (Ueda & Yoza, 2009). Bis(tetramethylammonium)bis[2-[4,5-bis(methylsulfanyl)-1,3-dithiol-2- ylidene]-1,3-dithiole-4,5-bis(thiolato)]zinc (402.4 mg, 0.4322 mmol) was reacted with 4-iodo-2-methylsulfanyl-1,3-dithiole-2-ylium tetrafluoroborate (457.5 mg, 1.2639 mmol) in THF-DMF (5:1 = *v*/*v*) at room temperature under nitrogen. Stirring was carried out for 12 h. After separation of the reaction mixture by column chromatography on silica gel (eluent CS_2_) followed by recrystallization from CS_2_/hexane, (5*E*)-2-[4,5-bis(methylsulfanyl)-1,3-dithiole-2-ylidene]-5-(4-iodo- 1,3-dithiole-2-ylidene)-1,3-dithiolan-4-thione (II) was obtained as dark-green needles in 74% yield.

When compound (II) (151.6 mg, 0.2733 mmol) was reacted with mercury(II) acetate (191.6 mg, 0.6012 mmol) in THF-AcOH (50:1 = *v*/*v*), compound (I) was obtained as dark-red platelets in 58% yield by recrystallization from CS_2_/hexane.

### Refinement

The H atoms were geometrically placed with C—H = 0.95-0.98 Å, and refined as in the riding model approximation with *U*_iso_(H)= 1.2-1.5*U*_eq_(C).

The maximum and minimum residual electron density peaks of 2.34 and -1.13 eÅ^-3^, respectively, were located 1.00 Å and 0.83 Å from the I1 atom, respectively.

### Crystal data

**Table:**

C_11_H_7_IOS_8_	*Z* = 2
*M**_r_* = 538.55	*F*(000) = 524
Triclinic, *P*1	*D*_x_ = 2.109 Mg m^−^^3^
Hall symbol: -P 1	Mo *K*α radiation, λ = 0.71073 Å
*a* = 8.309 (3) Å	Cell parameters from 2153 reflections
*b* = 8.344 (3) Å	θ = 2.8–25.3°
*c* = 14.618 (7) Å	µ = 2.87 mm^−^^1^
α = 90.851 (6)°	*T* = 93 K
β = 105.132 (6)°	Block, dark-red
γ = 118.510 (4)°	0.04 × 0.04 × 0.04 mm
*V* = 848.0 (6) Å^3^	

### Data collection

**Table:**

Bruker APEXII CCD area-detector diffractometer	3820 independent reflections
Radiation source: Bruker TXS fine-focus rotating anode	3065 reflections with *I* > 2σ(*I*)
Bruker Helios multilayer confocal mirror	*R*_int_ = 0.050
Detector resolution: 8.333 pixels mm^-1^	θ_max_ = 27.5°, θ_min_ = 1.5°
φ and ω scans	*h* = −10→10
Absorption correction: multi-scan (*SADABS*; Sheldrick, 1996)	*k* = −10→10
*T*_min_ = 0.894, *T*_max_ = 0.894	*l* = −18→18
9773 measured reflections	

### Refinement

**Table:**

Refinement on *F*^2^	Primary atom site location: structure-invariant direct methods
Least-squares matrix: full	Secondary atom site location: difference Fourier map
*R*[*F*^2^ > 2σ(*F*^2^)] = 0.055	Hydrogen site location: inferred from neighbouring sites
*wR*(*F*^2^) = 0.161	H-atom parameters constrained
*S* = 1.07	*w* = 1/[σ^2^(*F*_o_^2^) + (0.0842*P*)^2^ + 3.4063*P*] where *P* = (*F*_o_^2^ + 2*F*_c_^2^)/3
3820 reflections	(Δ/σ)_max_ = 0.001
190 parameters	Δρ_max_ = 2.34 e Å^−^^3^
0 restraints	Δρ_min_ = −1.13 e Å^−^^3^

### Special details

**Table:**

Geometry. All e.s.d.'s (except the e.s.d. in the dihedral angle between two l.s. planes) are estimated using the full covariance matrix. The cell e.s.d.'s are taken into account individually in the estimation of e.s.d.'s in distances, angles and torsion angles; correlations between e.s.d.'s in cell parameters are only used when they are defined by crystal symmetry. An approximate (isotropic) treatment of cell e.s.d.'s is used for estimating e.s.d.'s involving l.s. planes.
Refinement. Refinement of *F*^2^ against ALL reflections. The weighted *R*-factor *wR* and goodness of fit *S* are based on *F*^2^, conventional *R*-factors *R* are based on *F*, with *F* set to zero for negative *F*^2^. The threshold expression of *F*^2^ > σ(*F*^2^) is used only for calculating *R*-factors(gt) *etc*. and is not relevant to the choice of reflections for refinement. *R*-factors based on *F*^2^ are statistically about twice as large as those based on *F*, and *R*- factors based on ALL data will be even larger.

### Fractional atomic coordinates and isotropic or equivalent isotropic displacement parameters (Å^2^)

**Table:**

	*x*	*y*	*z*	*U*_iso_*/*U*_eq_	
C1	0.7414 (11)	0.3494 (11)	1.2804 (5)	0.0208 (15)	
C2	0.7832 (11)	0.2717 (10)	1.2163 (5)	0.0200 (15)	
H2A	0.7957	0.1652	1.2252	0.024*	
C3	0.7600 (10)	0.5445 (10)	1.1458 (5)	0.0171 (14)	
C4	0.7523 (10)	0.6656 (10)	1.0854 (5)	0.0147 (14)	
C5	0.7096 (11)	0.8045 (10)	1.1130 (5)	0.0176 (15)	
C6	0.7518 (10)	0.8433 (10)	0.9411 (5)	0.0188 (15)	
C7	0.7691 (10)	0.9104 (10)	0.8586 (5)	0.0168 (14)	
C8	0.7811 (11)	1.1023 (10)	0.7195 (5)	0.0195 (15)	
C9	0.8259 (11)	0.9774 (10)	0.6922 (6)	0.0227 (16)	
C10	0.5811 (14)	1.2907 (13)	0.6735 (8)	0.040 (2)	
H10A	0.5663	1.3860	0.6401	0.060*	
H10B	0.6051	1.3221	0.7424	0.060*	
H10C	0.4633	1.1708	0.6481	0.060*	
C11	0.7773 (12)	0.7290 (12)	0.5471 (7)	0.0317 (19)	
H11A	0.8072	0.7093	0.4888	0.048*	
H11B	0.6381	0.6763	0.5324	0.048*	
H11C	0.8218	0.6686	0.5964	0.048*	
I1	0.72165 (7)	0.26967 (7)	1.41327 (4)	0.02158 (17)	
O1	0.6819 (8)	0.8263 (7)	1.1898 (4)	0.0248 (12)	
S2	0.6928 (3)	0.9427 (3)	1.02369 (13)	0.0204 (4)	
S3	0.7856 (3)	0.6574 (3)	0.97138 (13)	0.0192 (4)	
S4	0.7150 (3)	0.5414 (2)	1.25643 (13)	0.0180 (4)	
S5	0.8134 (3)	0.3773 (3)	1.11542 (14)	0.0223 (4)	
S6	0.7799 (3)	1.2779 (3)	0.65508 (14)	0.0222 (4)	
S7	0.8283 (3)	0.8205 (3)	0.77148 (14)	0.0215 (4)	
S8	0.7337 (3)	1.0982 (3)	0.83040 (14)	0.0212 (4)	
S9	0.8974 (4)	0.9749 (3)	0.59090 (16)	0.0318 (5)	

### Atomic displacement parameters (Å^2^)

**Table:**

	*U*^11^	*U*^22^	*U*^33^	*U*^12^	*U*^13^	*U*^23^
C1	0.018 (4)	0.025 (4)	0.019 (4)	0.011 (3)	0.004 (3)	0.005 (3)
C2	0.029 (4)	0.017 (4)	0.018 (4)	0.015 (3)	0.008 (3)	0.006 (3)
C3	0.014 (3)	0.015 (3)	0.021 (4)	0.005 (3)	0.006 (3)	0.001 (3)
C4	0.018 (3)	0.014 (3)	0.016 (3)	0.010 (3)	0.006 (3)	0.004 (3)
C5	0.021 (4)	0.021 (4)	0.014 (3)	0.011 (3)	0.007 (3)	0.004 (3)
C6	0.019 (4)	0.020 (4)	0.018 (4)	0.010 (3)	0.006 (3)	0.003 (3)
C7	0.017 (3)	0.017 (4)	0.014 (3)	0.007 (3)	0.005 (3)	0.002 (3)
C8	0.022 (4)	0.021 (4)	0.014 (4)	0.008 (3)	0.010 (3)	0.005 (3)
C9	0.024 (4)	0.016 (4)	0.027 (4)	0.005 (3)	0.014 (3)	0.007 (3)
C10	0.045 (6)	0.035 (5)	0.062 (7)	0.028 (5)	0.032 (5)	0.027 (5)
C11	0.030 (4)	0.026 (4)	0.038 (5)	0.010 (4)	0.016 (4)	0.002 (4)
I1	0.0234 (3)	0.0216 (3)	0.0216 (3)	0.0115 (2)	0.0088 (2)	0.00788 (19)
O1	0.034 (3)	0.022 (3)	0.026 (3)	0.017 (3)	0.014 (3)	0.010 (2)
S2	0.0297 (10)	0.0244 (10)	0.0164 (9)	0.0194 (8)	0.0092 (8)	0.0063 (7)
S3	0.0267 (10)	0.0213 (9)	0.0160 (9)	0.0154 (8)	0.0094 (8)	0.0053 (7)
S4	0.0220 (9)	0.0180 (9)	0.0191 (9)	0.0113 (7)	0.0111 (7)	0.0063 (7)
S5	0.0311 (10)	0.0206 (9)	0.0213 (10)	0.0160 (8)	0.0110 (8)	0.0041 (7)
S6	0.0270 (10)	0.0246 (10)	0.0191 (9)	0.0134 (8)	0.0119 (8)	0.0101 (7)
S7	0.0282 (10)	0.0221 (9)	0.0194 (9)	0.0145 (8)	0.0113 (8)	0.0048 (7)
S8	0.0267 (10)	0.0261 (10)	0.0185 (9)	0.0169 (8)	0.0109 (8)	0.0084 (7)
S9	0.0504 (13)	0.0232 (10)	0.0270 (11)	0.0150 (10)	0.0265 (10)	0.0058 (8)

### Geometric parameters (Å, °)

**Table:**

C1—C2	1.337 (11)	C7—S7	1.755 (7)
C1—S4	1.746 (8)	C7—S8	1.762 (8)
C1—I1	2.083 (8)	C8—C9	1.349 (11)
C2—S5	1.741 (8)	C8—S6	1.755 (8)
C2—H2A	0.9500	C8—S8	1.763 (7)
C3—C4	1.365 (10)	C9—S9	1.736 (8)
C3—S5	1.737 (7)	C9—S7	1.765 (8)
C3—S4	1.751 (8)	C10—S6	1.792 (9)
C4—C5	1.444 (10)	C10—H10A	0.9800
C4—S3	1.764 (7)	C10—H10B	0.9800
C5—O1	1.230 (9)	C10—H10C	0.9800
C5—S2	1.777 (8)	C11—S9	1.813 (9)
C6—C7	1.350 (10)	C11—H11A	0.9800
C6—S2	1.747 (8)	C11—H11B	0.9800
C6—S3	1.747 (8)	C11—H11C	0.9800
			
C2—C1—S4	118.7 (6)	C8—C9—S9	124.2 (6)
C2—C1—I1	124.9 (6)	C8—C9—S7	116.2 (6)
S4—C1—I1	116.2 (4)	S9—C9—S7	119.4 (5)
C1—C2—S5	116.1 (6)	S6—C10—H10A	109.5
C1—C2—H2A	122.0	S6—C10—H10B	109.5
S5—C2—H2A	122.0	H10A—C10—H10B	109.5
C4—C3—S5	121.0 (6)	S6—C10—H10C	109.5
C4—C3—S4	124.0 (6)	H10A—C10—H10C	109.5
S5—C3—S4	115.1 (4)	H10B—C10—H10C	109.5
C3—C4—C5	119.7 (7)	S9—C11—H11A	109.5
C3—C4—S3	122.9 (6)	S9—C11—H11B	109.5
C5—C4—S3	117.3 (5)	H11A—C11—H11B	109.5
O1—C5—C4	124.7 (7)	S9—C11—H11C	109.5
O1—C5—S2	121.3 (6)	H11A—C11—H11C	109.5
C4—C5—S2	113.9 (5)	H11B—C11—H11C	109.5
C7—C6—S2	120.1 (6)	C6—S2—C5	96.5 (4)
C7—C6—S3	123.1 (6)	C6—S3—C4	95.3 (3)
S2—C6—S3	116.8 (4)	C1—S4—C3	94.2 (4)
C6—C7—S7	123.5 (6)	C3—S5—C2	95.8 (4)
C6—C7—S8	121.9 (6)	C8—S6—C10	102.2 (4)
S7—C7—S8	114.6 (4)	C7—S7—C9	96.0 (4)
C9—C8—S6	124.3 (6)	C7—S8—C8	95.1 (4)
C9—C8—S8	118.1 (6)	C9—S9—C11	101.7 (4)
S6—C8—S8	117.4 (4)		
